# Supplementary material for: MiR-221-3p/222-3p Cluster Expression in Human Adipose Tissue Is Related to Obesity and Type 2 Diabetes
Source: Int J Mol Sci. 2023 Dec 13;24(24):17449. doi: 10.3390/ijms242417449 (PMC10744326; doi:10.3390/ijms242417449)
Supplement: Supplementary file 1 [file ijms-24-17449-s001.zip › ijms-2699438-supplementary.pdf]

SUPPLEMENTARY TABLE

Table S1. Gene aliases and names

VALIDATED GENE	NAME
ABLIM1	Actin-binding LIM protein 1
ACTB	Actin, cytoplasmic 1
ACTG1	Actin, cytoplasmic 2
ADAM1A	Adam Metallopeptidase Domain 1A (Pseudogene)
ARHGAP42	Rho GTPase-activating protein 42
C19orf12	Protein C19orf12
CABYR	Calcium-binding tyrosine phosphorylation-regulated protein
CDKN1C	Cyclin-dependent kinase inhibitor 1C
CHORDC1	Cysteine and histidine-rich domain-containing protein 1
CIAPIN1	Anamorsin
COG5	Conserved oligomeric Golgi complex subunit 5
CORO1A	Coronin-1A
CPSF6	Cleavage and polyadenylation specificity factor subunit 6
CSTF2T	Cleavage stimulation factor subunit 2 tau variant
DDIT4	DNA damage-inducible transcript 4 protein
DDX6	Probable ATP-dependent RNA helicase DDX6
DERL2	Derlin-2
DIRAS3	GTP-binding protein Di-Ras3
DKK2	Dickkopf-related protein 2
ETS1	Protein C-ets-1
EXOC8	Exocyst complex component 8
FAM35A	Protein FAM35A
FAM84A	Protein FAM84A
FBXL18	F-box/LRR-repeat protein 18
FOS	Proto-oncogene c-Fos
GCN1L1	Alternative protein GCN1L1
HNRNPD	Heterogeneous nuclear ribonucleoprotein D0
KPNA6	Importin subunit alpha-7
MAT2A	S-adenosylmethionine synthase isoform type-2
MGMT	Methylated-DNA--protein-cysteine methyltransferase
MKKS	McKusick-Kaufman/Bardet-Biedl syndromes putative chaperonin
OBFC1	CST complex subunit STN1
OIP5	Protein Mis18-beta
PGPEP1	Pyroglutamyl-peptidase 1
PIWIL1	Piwi-like protein 1
PRPS1L1	Ribose-phosphate pyrophosphokinase 3
RBMS2	RNA-binding motif, single-stranded-interacting protein 2
RNF4	E3 ubiquitin-protein ligase RNF4

SAPCD2	Suppressor APC domain-containing protein 2
SELE	E-selectin
SLC25A36	Solute carrier family 25 member 36
SMC2	Structural maintenance of chromosomes protein 2
SNX4	Sorting nexin-4
STAT5A	Signal transducer and activator of transcription 5A
TIMP3	Metalloproteinase inhibitor 3
TIPARP	TCDD-inducible poly [ADP-ribose] polymerase
TMCC1	Transmembrane and coiled-coil domains protein 1
TNFSF10	Tumor necrosis factor ligand superfamily member 10
TRAT1	T-cell receptor-associated transmembrane adapter 1
TSPAN13	Tetraspanin-13
ZEB2	Zinc finger E-box-binding homeobox 2
ZFP30	Zinc finger protein 30 homolog
ZNF236	Zinc finger protein 236
**NON VALIDATED GENE**	**NAME**
AKT3	RAC-gamma serine/threonine-protein kinase
APAF1	Apoptotic protease-activating factor 1
BCL2	Apoptosis regulator Bcl-2
CASP10	Caspase 10
CELF1	CUGBP Elav-like family member 1
CREB1	Cyclic AMP-responsive element-binding protein 1
DVL2	Segment polarity protein dishevelled homolog DVL-2
IKBKG	NF-kappa-B essential modulator
IL1RAP	Interleukin-1 receptor accessory protein
LIFR	Leukemia inhibitory factor receptor
NCK1	Cytoplasmic protein NCK1
PDGFD	Platelet-derived growth factor D
PIK3CD	Phosphatidylinositol 4,5-bisphosphate 3-kinase catalytic subunit delta isoform
PPP3R1	Calcineurin subunit B type 1
PRKCB	Protein kinase C beta type
STAT2	Signal transducer and activator of transcription 2
TNFSF10	Tumor necrosis factor ligand superfamily member 10

Table S2. Binding sites of miR-221-3p, miR-222-3p within the objective.

GENE	ID	3' UTR length	miRNA	Position	seed match	Context ++ score	Context ++ score Percentil	Pct
**ETS1**	ENST00000531611.1	3774	hsa-miR-221-3p	3581-3587	7mer-m8	-0.09	65	0.10
**ETS1**	ENST00000531611.1	3774	hsa-miR-222-3p	3581-3587	7mer-m8	-0.11	70	0.1

**ETS1**	ENST00000531611.1	3774	hsa-miR-221-3p	1231-1237	7mer-1A	-0.09	65	<0.1
**ETS1**	ENST00000531611.1	3774	hsa-miR-222-3p	1231-1237	7mer-1A	-0.1	67	<0.1
**ETS1**	ENST00000345075.4	3600	hsa-miR-221-3p	3403-3409	7mer-m8	-0.08	62	0.10
**ETS1**	ENST00000345075.4	3600	hsa-miR-222-3p	3403-3409	7mer-m8	-0.10	68	0.10
**ETS1**	ENST00000345075.4	3600	hsa-miR-221-3p	3403-3409	7mer-1A	-0.08	64	<0.1
**ETS1**	ENST00000345075.4	3600	hsa-miR-222-3p	3403-3409	7mer-1A	-0.10	67	<0.1
**IL1RAP**	ENST00000422485.1	3005	hsa-miR-221-3p	199-205	7mer-m8	-0.28	94	<0.1
**IL1RAP**	ENST00000422485.1	3005	hsa-miR-222-3p	199-205	7mer-m8	-0.29	94	<0.1
**IL1RAP**	ENST00000317757.3	1447	hsa-miR-221-3p	865-871	7mer-m8	-0.07	56	<0.1
**IL1RAP**	ENST00000317757.3	1447	hsa-miR-222-3p	865-871	7mer-m8	-0.1	67	<0.1
**DVL2**	ENST00000005340.5	524	hsa-miR-221-3p	449-455	7mer-m8	-0.11	70	<0.1
**DVL2**	ENST00000005340.5	524	hsa-miR-222-3p	449-455	7mer-m8	-0.12	72	<0.1

Table S3. Applied Biosystem' TaqMan probes

Gene	Name	Dye	**TaqMan Assay ID**	Amplicon	Ref. sequence
**PPIA**	*Cyclophilin A*	VIC	Hs_99999904_m1	98	NM_021130.3
**Dvl2**	*Dishevelled segment polarity protein 2*	FAM	Hs_1005253_m1	55	NM_004422.2
**Ets1**	*ETS proto-oncogene 1, transcription factor*	FAM	Hs_00428293_m1	99	NM_001143820.1
**Il1rap**	Interleukin 1 receptor accessory protein	FAM	Hs_00370509_m1	113	NM_001167928.1

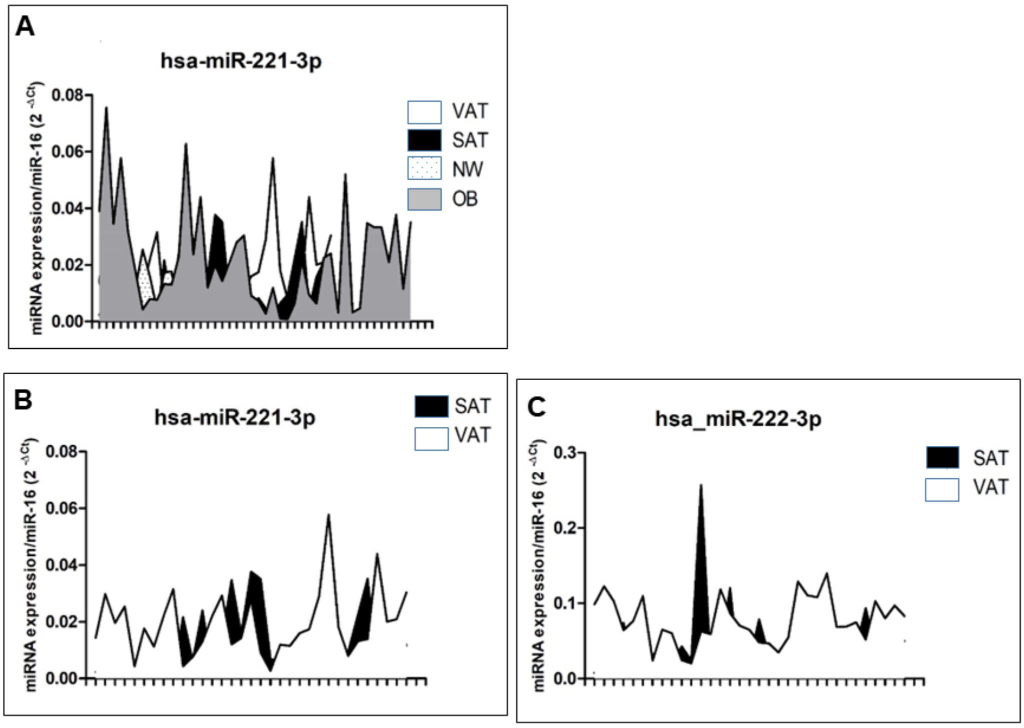

Figure S1. Regression analysis of miR-221-3p/222-3p Cluster in Human VAT and SAT. MiRNAs were extracted from VAT and SAT, and their expression levels were measured via qPCR. Relative quantification of expression levels was performed using the comparative threshold cycle (Ct) method, with Hsa-miR-16 serving as the endogenous control. Data are presented as the mean ± SEM. The data underwent analysis via a multivariate general linear model, incorporating tissue groups (VAT vs. SAT), diabetes status (NG vs. T2D subjects), obesity status (NW vs. OB) and sex (male vs. female) as independent variables, with the expression levels of miR-221-3p and miR-222-3p as dependent variables and HDLc and age as co-variates. A linear regression analysis was done.

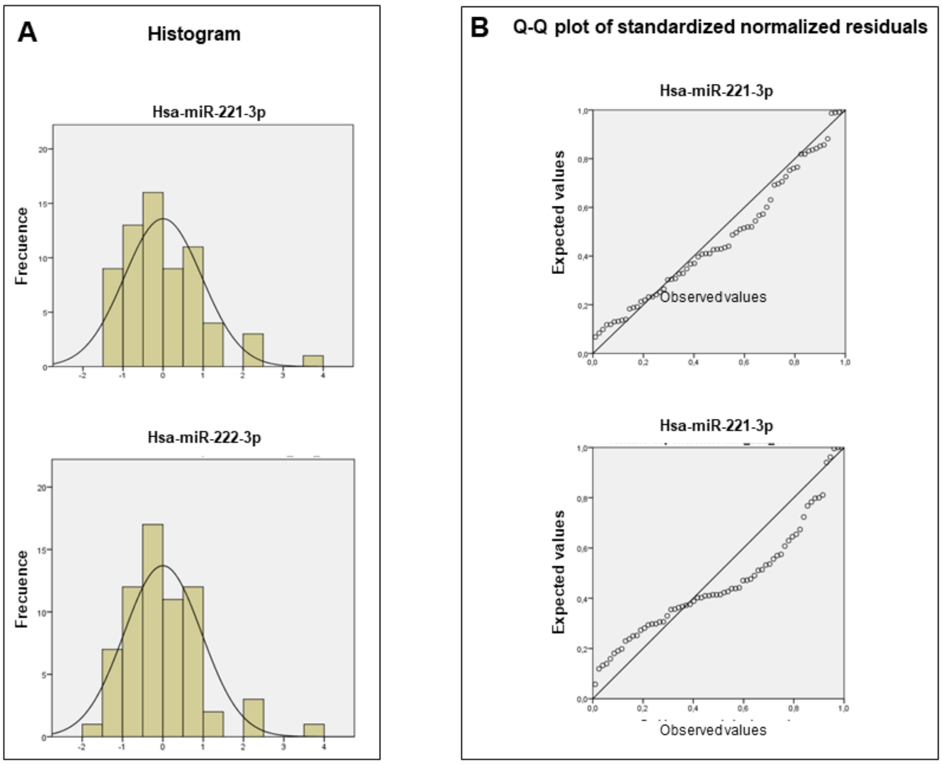

Figure S2. Regression analysis of miR-221-3p/222-3p Cluster in Human VAT and SAT. MiRNAs were extracted from VAT and SAT, and their expression levels were measured via qPCR. Relative quantification of expression levels was performed using the comparative threshold cycle (Ct) method, with Hsa-miR-16 serving as the endogenous control. Data are presented as the mean ± SEM. The data underwent analysis via a multivariate general linear model, incorporating tissue groups (VAT vs. SAT), diabetes status (NG vs. T2D subjects), obesity status (NW vs. OB) and sex (male vs. female) as independent variables, with the expression levels of miR-221-3p and miR-222-3p as dependent variables and HDLc and age as co-variates. A linear regression analysis was done. The Histogram (S2A) and QQ-plot (S2B) show that all parameters tested have a normal distribution.

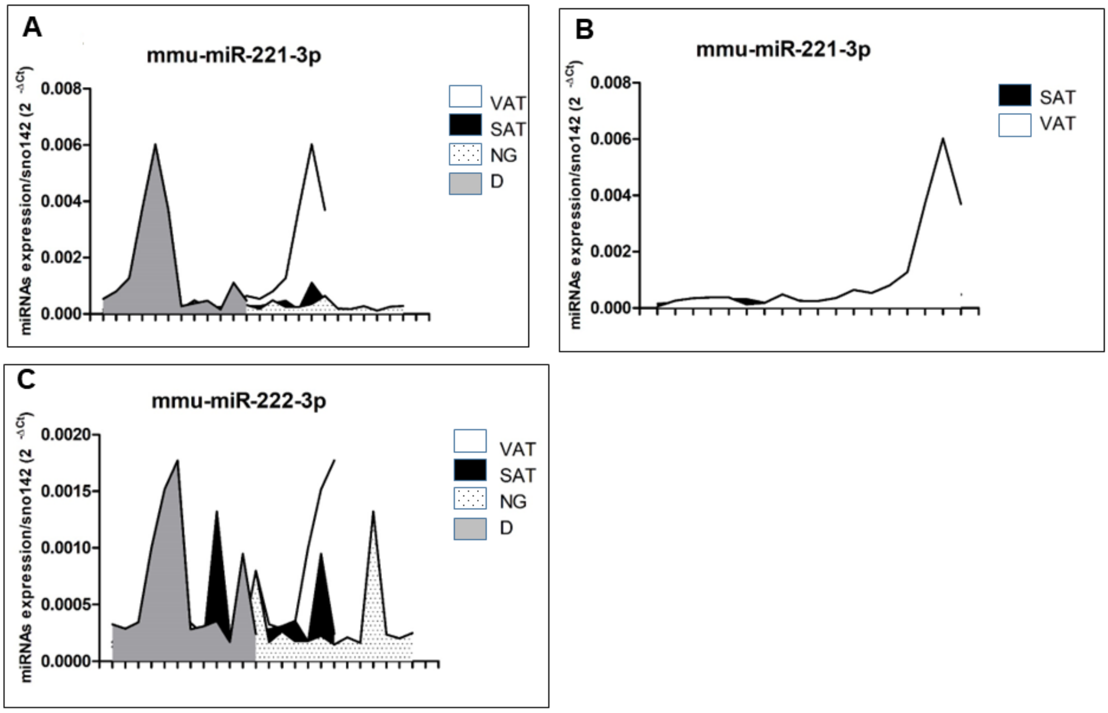

Figure S3. Regression analysis of miR-221-3p/222-3p Cluster in Mouse VAT and SAT. MiRNAs were extracted from VAT and SAT, and their expression levels were measured via qPCR. Relative quantification of expression levels was performed using the comparative threshold cycle (Ct) method, with SnoRNA 142 serving as the endogenous control. Data are presented as the mean ± SEM. The data underwent analysis via a multivariate general linear model, incorporating tissue groups (VAT vs. SAT), diabetes status (NG vs. T2D subjects) and obesity status (NW vs. OB) as independent variables, with the expression levels of miR-221-3p and miR-222-3p as dependent variables A linear regression analysis was done.

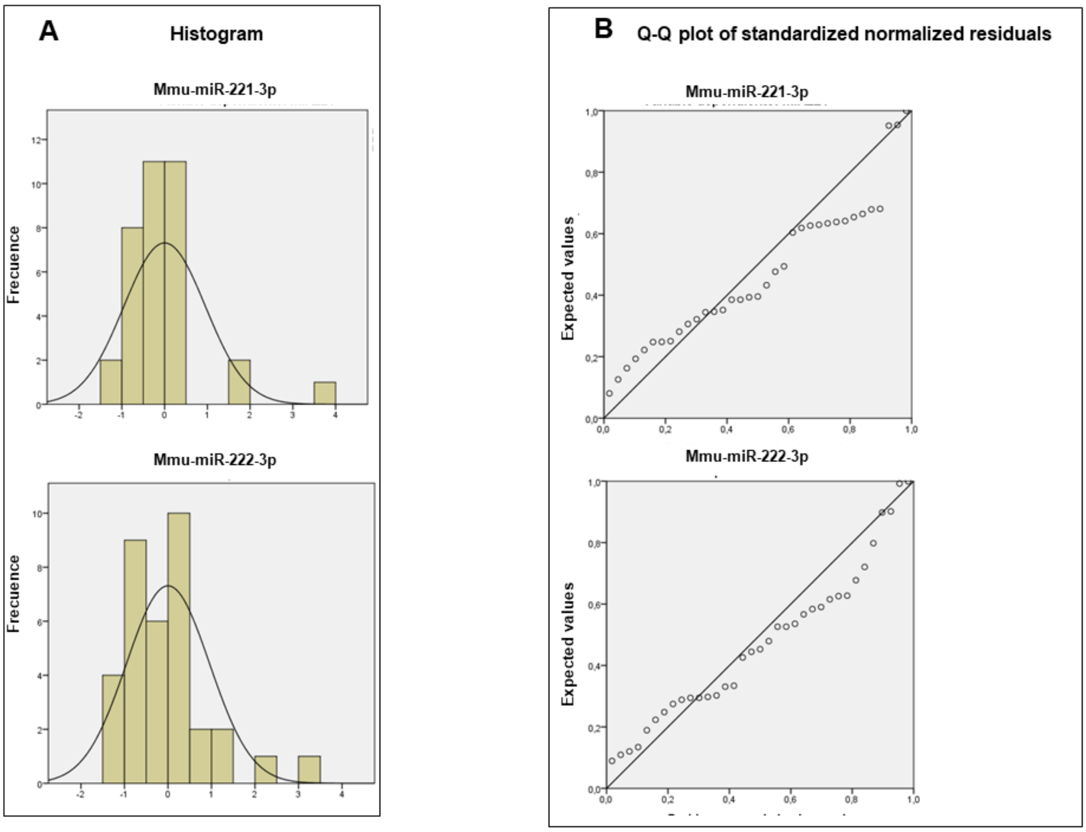

Figure S4. Regression analysis of miR-221-3p/222-3p Cluster in Mouse VAT and SAT. MiRNAs were extracted from VAT and SAT, and their expression levels were measured via qPCR. Relative quantification of expression levels was performed using the comparative threshold cycle (Ct) method, with SnoRNA 142 serving as the endogenous control. Data are presented as the mean ± SEM. The data underwent analysis via a multivariate general linear model, incorporating tissue groups (VAT vs. SAT), diabetes status (NG vs. T2D subjects) and obesity status (NW vs. OB) as independent variables, with the expression levels of miR-221-3p and miR-222-3p as dependent variables A linear regression analysis was done. The Histogram (S4A) and QQ-plot (S4B) show that all parameters tested have a normal distribution.

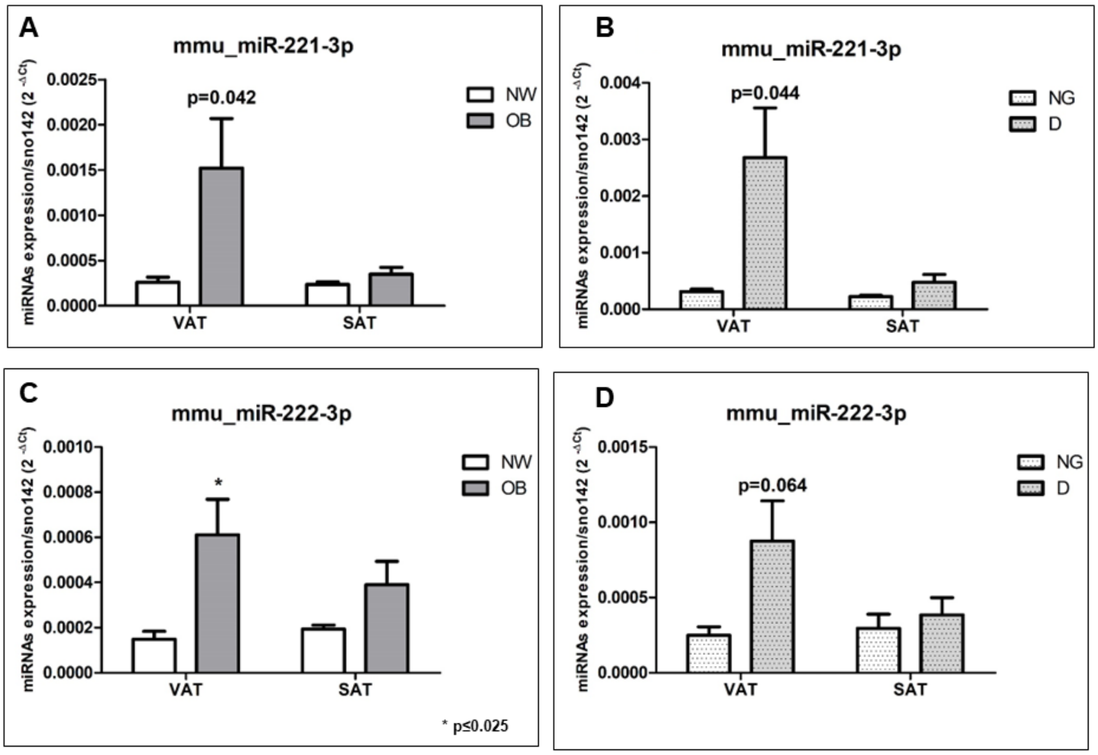

Figure S5. Expression Profiles of miR-221-3p/222-3p Cluster in Mouse VAT and SAT. MiRNAs were isolated from VAT and SAT, and their gene expression levels were assessed via qPCR. Relative quantification of the expression levels was conducted using the comparative threshold cycle (Ct) method, with SnoRNA142 serving as the endogenous control. Data (n=18) are presented as the mean ± SEM. The data underwent analysis via Student's t test, incorporating diabetes status (NG vs. type 2 diabetic mice), and obesity status (NW vs. OB) for different tissue type (VAT and SAT). Pairwise comparisons were used to show the effect obesity, and diabetes in each tissue group. *p<0.025 shows a significant difference.
